# The validity of self-reported body mass index in a population-based osteoarthritis study

**DOI:** 10.1186/1471-2474-15-442

**Published:** 2014-12-17

**Authors:** Karin Magnusson, Ida K Haugen, Nina Østerås, Lars Nordsletten, Bård Natvig, Kåre Birger Hagen

**Affiliations:** National Advisory Unit on Rehabilitation in Rheumatology, Department of Rheumatology, Diakonhjemmet Hospital, Post box 23, Vinderen, 0319 Oslo Norway; Department of Rheumatology, Diakonhjemmet Hospital, Post box 23, Vinderen, 0319 Oslo, Norway; Orthopaedic Department, Oslo University Hospital, 0407 Oslo Norway; Department of General Practice, Institute of Health and Society, University of Oslo, Post box 1130, Blindern, 0318 Oslo Norway; Department of Health Sciences, Institute of Health and Society, University of Oslo, Post box 1089, Blindern, 0317 Oslo Norway

**Keywords:** Body mass index, Obesity, Self-report, Osteoarthritis, Age, Validity

## Abstract

**Background:**

Obesity is a well-known risk factor for osteoarthritis (OA). The majority of obesity research in OA is performed using self-reported BMI-data, however, its validity in persons with OA is unknown. The aim of this study was to compare the validity of self-reported body mass index (BMI) in persons with and without clinical osteoarthritis (OA) in a population-based survey.

**Methods:**

Height and weight were self-reported, and thereafter measured in 600 persons with and without clinical OA according to the American College of Rheumatology-criteria (knees, hips and/or hands). We compared the differences between measured and self-reported heights, weights and BMIs (kg/m^2^) for the two groups and explored whether demographic/clinical factors were associated with inaccurate reporting in the OA patients using multivariate linear regression analyses.

**Results:**

Mean (SD) age was 64 (8.7) years and 412 (69%) were women. Participants with clinical OA (n = 449) underreported their BMI to a greater extent than participants without clinical OA (n = 151) [mean (SD) difference 1.34 (1.68) kg/m^2^ and 0.78 (1.40) kg/m^2^ (p = 0.000), respectively]. There was a strong dose-dependent association between higher measured BMI and greater underreporting of BMI in multivariate analyses (BMI 25–29.99 kg/m^2^: B = 0.40, 95% CI, 0.06, 0.77), BMI ≥ 30 kg/m^2^: B = 1.30, 95% CI, 0.86, 1.75) in the clinical OA patients. A higher age as well as the time interval from self-reported to measured BMI-data were associated with inaccurate reporting.

**Conclusions:**

Researchers using self-reported height and weight data should be aware of limited agreement with actual height and weight in overweight and obese individuals with clinical OA.

## Background

Obesity is one of very few modifiable risk factors for lower limb osteoarthritis (OA)[1, 2] and may also be a risk factor for hand OA[3]. Measures of overweight and obesity are frequently based on self-reported height and weight and reported as Body Mass Index (BMI)[1, 2]. However, the self-reported values may reflect people’s wishes and desires rather than reality[4], and results from studies using self-reported BMI may therefore be biased.

Using self-reported rather than measured BMI in research will save much time and costs. However, a systematic review of the validity of self-reported height and weight showed an overall trend of underestimating weight and overestimating height across a wide range of different populations[5]. The great individual variability made it impossible to pool the results and to make an accurate estimation of the actual BMI based on the self-reported BMI. The review emphasized the importance of estimating the extent of misclassification in each specific patient group. Previous studies have shown that the reliability of self-reported BMI-data may be dependent on age, sex, socioeconomic status and measured BMI category[4, 6, 7]. The differences in health outcomes between persons with a high and low socioeconomic status are often due to modifiable lifestyle factors or due to poor mental health[8]. Hence, lifestyle factors and health-related variables might be hypothesized to play a role for inaccurately reported BMI-data.

In OA populations, the trustworthiness of self-reported BMI and the characteristics of inaccurate reporters are unknown. As obesity is one of very few known modifiable risk factors for both incident and progressive OA, obesity research may have a significant impact on future prevention and disease management. Improved knowledge of the trustworthiness of self-reported BMI data in OA patients is important for the design and interpretation of future studies and may shed new light on existing studies of obesity in OA.

Hence, the aims of this study were to compare the validity of self-reported heights, weights and BMIs in persons with and without clinical OA and to study factors associated with inaccurate reporting of these data in persons with clinical OA.

## Methods

This cross-sectional study is a part of the Musculoskeletal pain in Ullensaker study, where all persons aged 40–79 years in 2010 living in Ullensaker municipality were sent questionnaires regarding musculoskeletal complaints (n = 12.155 of whom 4994 responded (41%)). Height (cm) and weight (kg) were self-reported on the questionnaire with participants being unaware of later measurement. Those who answered "Yes" to the question "Do you have osteoarthritis in the knees, hips and/or hand?" were asked to attend a clinical examination at Diakonhjemmet Hospital, Oslo, Norway. We measured height (cm) and weight (kg) with the participant wearing light indoor clothing, shoes removed and pockets emptied and screened for clinical OA in the knees, hips and hands. A detailed protocol of the study has been published elsewhere[9]. Approval for the study was granted by the Norwegian Regional Committee for Medical and Health Research Ethics (Ref. no. 2008/812a) and the Norwegian Data Inspectorate, and all participants signed informed consent.

### BMI

BMI was calculated based on both self-reported and measured height and weight (kg/m^2^). Heights in centimetres and weight in kilograms were both measured once by different project coordinators in a standardized way. We have no data on reliability. When we refer to "self-reported BMI", we mean calculated BMI based on self-reported height and weight. Similarly, when we refer to "measured BMI" we mean calculated BMI based on measured height and weight. Participants were divided into three BMI-categories (measured BMI) according to the World Health Organization[10]. Normal weight was defined as BMI <24.99 kg/m^2^, overweight as BMI 25–29.99 kg/m^2^ and obesity as BMI ≥30 kg/m^2^.

### OA variables

A rheumatologist or medical students screened for clinical OA in the knees, hips and hands according to the American College of Rheumatology (ACR) criteria[11]. Participants were tested only once and no reliability data exist. Those who fulfilled the criteria in either the knees, hips and/or hands were classified as having a clinically meaningful OA diagnosis, whereas those not fulfilling the criteria in any joint were classified as having no clinical OA.

### Covariates

The time interval between the date of self-reporting BMI-data and the date of clinical examination was measured in months. Educational status was used as a measure of socioeconomic status and defined as the highest education level achieved. It was categorized into "primary/upper secondary school" versus "≥1 year at college/university". We used the International Physical Activity Questionnaire score for measuring physical activity level (0–2 scale representing low, moderate and high levels)[12]. Smoking status was dichotomized into "never/former smoker" versus "present smoker". The educational status and smoking covariates were dichotomized in order to improve statistical efficiency. This was not done with IPAQ as it is measured on a validated questionnaire with a standardized categorization. Mental health status was measured by the Short-Form (SF)-36 mental summary component score (0–100, higher score = better health)[13].

### Statistics

The differences between self-reported and measured BMI-data were calculated. Close agreement was defined as a difference of +/- 1.00-1.99 kg/m^2^, whereas exact agreement was defined as a difference of +/- 0.99 kg/m^2^ or less. A difference of above +/- 2.00 kg/m^2^ was classified as poor agreement. We examined the percentages agreement between measured and self-reported BMI within each BMI-category and compared participants with and without clinical OA using Chi-square test or Fischer’s exact test. We also examined the absolute differences in BMI, heights and weights across measured BMI-categories for participants with and without clinical OA and compared the groups using independent sample t-tests after having inspected data for normality (examining histograms). A positive difference indicates underreporting (self-reported < measured) and a negative difference indicates overreporting (self-reported > measured).

In multivariate linear regression analyses (robust standard error), we explored whether demographic and clinical factors were associated with the difference between self-reported and measured data for the participants with clinical OA taking the time interval between self-report and measurement into account. We used a descriptive modelling approach not aimed at either explanation or prediction.

P-values ≤0.05 were considered statistically significant. All analyses were performed using STATA IC13.

## Results

In total, 1049 participants had self-reported OA in the knees, hips and/or hands, of whom 1019 were asked to attend the clinical examination and signed informed consent in the time interval between self-report and measurement. Of the 630 participants who attended the examination, we excluded n = 19 participants due to missing self-reported BMI-data and n = 13 participants due to missing clinical joint examination (of whom 2 also had missing BMI-data). Reasons for not being willing to attend the clinical examination are unclear. Our study sample consisted of 600 persons, of whom 449 (74.8%) had clinical OA in the knees, hips and/or hands according to the ACR-criteria, and 151 (25.2%) who did not fulfil the ACR criteria for any of the three joint locations. Participants’ characteristics are presented in Table 1.

**Table 1:** **Participants' characteristics**

	Participants	Participants
	With OA*	Without OA^
	n = 449	n = 151
Sociodemographics and covariates		
Age, mean (SD)	64.6 (8.6)	61.8 (8.9)
Females, n (%)	321 (71.5)	91 (60.3)
Education status, n (%)		
primary/upper secondary school	318 (72.3)	98 (67.6)
>1 year college/university	122 (27.7)	47 (32.4)
IPAQ, low physical activity level, n (%)	112 (33.5)	37 (30.8)
Smoker, n (%)	64 (14.4)	21 (13.9)
SF-36 mental component score [0–100], mean (SD)	48.7 (10.9)	47.4 (10.9)
Months from self-report to measurement, mean (SD)	7.9 (4.3)	7.9 (4.0)
Anthropometrics		
Self-reported data		
Height (cm), mean (SD)	169.6 (8.6)	171.2 (9.2)
Weight (kg), mean (SD)	78.0 (15.7)	78.9 (16.1)
BMI (kg/m^2^), mean (SD)	27.0 (4.5)	26.8 (4.2)
Normalweight, n (%)	163 (36.3)	58 (38.4)
Overweight, n (%)	189 (42.1)	67 (44.4)
Obese, n (%)	97 (21.6)	26 (17.2)
Measured data		
Height (cm), mean (SD)	168.2 (8.6)	170.0 (9.3)
Weight (kg), mean (SD)	80.4 (16.4)	80.0 (16.3)
BMI (kg/m^2^), mean (SD)	28.3 (4.9)	27.6 (4.4)
Normalweight, n (%)	126 (28.1)	47 (31.1)
Overweight, n (%)	166 (37.0)	62 (41.1)
Obese, n (%)	157 (35.0)	42 (27.8)
OA site, n (%)		
Single site OA	285 (63.5)	
Knee OA	69 (15.4)	
Hip OA	42 (9.4)	
Hand OA	174 (38.8)	
Multisite OA	164 (36.5)	
Knees + hips	21 (4.7)	
Knees + hands	64 (14.3)	
Hips + hands	59 (13.1)	
Hips + knees + hands	20 (4.5)	

*Abbreviations*: *ACR* American college of Rheumatology, *BMI* Body Mass Index, *cm* centimeter, *IPAQ* International Physical Activity Questionnaire, *kg* kilograms, *m* meter, *OA* osteoarthritis, *SD* standard deviation, *SF-36* Short-Form 36. *Participants with clinical OA according to the ACR-criteria in the knees, hips and/or hands. ^ Participants without clinical OA according to the ACR-criteria in any joint.

Participants with clinical OA had less accurate self-reported BMI than participants without clinical OA (mean [SD] BMI difference 1.34 [1.68] kg/m^2^ and 0.78 [0.40] kg/m^2^ (p < 0.001), respectively). This was particularly evident for normalweight and obese participants (Table 2). For both participants with and without clinical OA, the percentage exact agreement was lower in those who were overweight or obese. Obese participants with clinical OA had significantly poorer agreement than obese participants without clinical OA, but no group differences in percentage agreement could be observed for normal- and overweight participants (Table 2).

**Table 2:** **Absolute differences and agreement between self-reported and measured BMI in participants with and without OA**

	Participants with clinical	Participants without clinical	P-value
	OA (n = 449)	OA (n = 151)	
Normalweight (<25 kg/m^2^)			
Difference between measured and self-reported BMI, mean (SD)	0.64 (0.93)	-0.00 (1.49)	0.000
Poor agreement, n (%)	3 (2.4)	2 (4.3)	
Close agreement, n (%)	42 (33.3)	16 (34.0)	
Exact agreement, n (%)	81 (64.3)	29 (61.7)	0.754
Overweight (25–29.99 kg/m^2^)			
Difference between measured and self-reported BMI, mean (SD)	1.20 (1.60)	1.05 (1.14)	0.256
Poor agreement, n (%)	18 (10.8)	3 (4.8)	
Close agreement, n (%)	89 (53.6)	35 (56.5)	
Exact agreement, n (%)	59 (35.5)	24 (38.7)	0.406
Obese (≥30 kg/m^2^)			
Difference between measured and self-reported BMI, mean (SD)	2.07 (1.95)	1.26 (1.31)	0.006
Poor agreement, n (%)	44 (28.0)	3 (7.1)	
Close agreement, n (%)	81 (51.6)	27 (64.3)	
Exact agreement, n (%)	32 (20.4)	12 (28.6)	0.010

*Abbreviations*: *BMI* Body Mass Index, *kg* kilograms, *m* meter, *OA* osteoarthritis. Poor agreement: ≥ ±2.00 kg/m^2^ difference, close agreement: ≥ ±1.00-1.99 kg/m^2^ difference, exact agreement: <±0-0.99 kg/m^2^ difference. Differences are the self-reported BMI (kg/m^2^) minus measured BMI (kg/m^2^). Positive values indicate underreporting, negative values indicate overreporting.

When looking at height and weight separately, we also observed a strong dose-dependent association between greater overreporting of height and underreporting of weight in those who were overweight or obese (data not shown). Participants with clinical OA underreported their weights to a greater extent than participants without clinical OA (mean (SD) difference in kg, 2.48 (0.20) and 1.13 (0.31), p < 0.001, respectively). In stratified analyses by BMI-category, the significant weight difference was only evident for the normalweight and obese participants, whereas for height, no group differences could be observed in any analyses (data not shown).

A dose-dependent association between a higher BMI-category and greater underreported BMI remained in multivariate analyses (Table 3). Furthermore, a higher age was associated with greater overreporting of height and underreporting of weight (Table 3).

**Table 3:** **Explanatory factors for differences between self-report and measurement in OA patients (n = 449)**

	Height difference	Weight difference	BMI difference
	(measured minus self-report)	(measured minus self-report)	(measured minus self-report)
	B (95% CI)	B (95% CI)	B (95% CI)
Age, mean (SD)	**-0.08 (-0.11, -0.05)**	**-0.07 (-0.13, -0.01)**	0.00 (-0.02, 0.03)
Sex, female	-0.17 (-0.75, 0.41)	0.23 (-0.81, 1.27)	0.33 (-0.06, 0.71)
BMI-category^a^			
Overweight (25–29.99 kg/m^2^)	-0.43 (-0.97, 0.11)	0.61 (-0.35, 1.57)	**0.42 (0.06, 0.77)**
Obese (≥30 kg/m^2^)	**-0.94 (-1.57, -0.31)**	**2.49 (1.34, 3.64)**	**1.30 (0.86, 1.75)**
Education status, >1 year college/university	-0.25 (-0.91, 0.42)	0.91 (-0.10, 1.91)	0.37 (-0.04, 0.77)
IPAQ, low physical activity level^b^			
Moderate activity	0.34 (-0.37, 1.06)	**-1.18 (-2.24, -0.13)**	**-0.54 (-0.96, -0.12)**
Vigorous activity	-0.06 (-0.64, 0.52)	-0.29 (-1.46, 0.87)	-0.11 (-0.55, 0.33)
Smoker	-0.44 (-1.11, 0.23)	-0.16 (-1.26, 0.94)	0.10 (-0.31, 0.51)
SF-36 mental component score [0–100]	0.01 (-0.01, 0.04)	0.01 (-0.03, 0.05)	-0.00 (-0.02, 0.01)
Time between self-report and measurement	**-0.15 (-0.23, -0.07)**	0.08 (-0.03, 0.20)	**0.08 (0.04, 0.12)**

*Abbreviatons*: *BMI* body mass index, *CI* confidence interval, *IPAQ* International Physical Activity Questionnaire, *SF-36* Short-Form 36. Multivariate linear regression analyses. Estimates are unstandardized. ^a^Normalweight is reference category ^b^Low physical activity level is reference category. Bold prints are significantly associated with the outcome.

## Discussion

This study showed a strong dose-dependent association between a higher measured BMI and greater overreporting of height and underreporting of weight and BMI both in persons with and without clinical OA. Participants with clinical OA reported their heights and weights less accurately than participants without clinical OA.

Although obesity is a known risk factor for knee OA[1, 2], the validity of obesity measured by self-report has to our knowledge not been previously studied in OA. Our results are in line with previous findings in other populations[5]. Furthermore, the findings extend existing knowledge as we showed a greater underreporting of BMI in persons with clinical OA compared to those without clinical OA. This may have consequences in the interpretation and design of existing and future epidemiological studies.

It is interesting that the participants with less accurate self-reported BMI share the same characteristics as those who report a greater burden of OA in previous studies. Similar to OA patients, more inaccurate reporters were older and more often obese[14]. The greater underreporting of BMI among OA patients may be due to higher mean weight, as heavier persons have more kilograms to be mistaken of. It may also be due to greater social desirability in women (who had more clinical OA)[4] as well as elderly likely forgetting the probable shrinkage in height that increases with age.

Higher age was associated with overreporting of both height and weight in the present study. These results are in line with a previous study by Kuczmarski *et al.* showing that self-reported heights and weights are reliable for younger persons, but may lead to severe misclassification when used for persons older than 60 years of age[6].

One limitation of the study is the time interval between completing the questionnaire (self-reported BMI) to clinical examination (measured BMI), and the risk of a true weight change during the period. However, larger time interval was associated with larger misreport of height but not weight. The larger misreport of height might be due to participants’ high age. Furthermore, all participants without clinical OA had self-reported OA. Participants with self-reported OA are likely to be different from persons with no self-reported OA. This might have influenced our results. However, with our approach using clinical OA criteria (including present OA pain) we were able to discriminate between participants having versus not having a clinically meaningful disease.

## Conclusion

In conclusion, our study showed that persons with clinically diagnosed OA underreported their BMI to a higher extent than people with no clinical OA. Furthermore, obese persons with diagnosed clinical OA were more likely to underreport than obese persons without clinical OA. Our findings should be taken into account in future OA studies of BMI based on self-report.

## Abbreviations

ACR: American college of rheumatology
BMI: Body mass index
OA: Osteoarthritis
SD: Standard deviation
SF-36: Short-form 36.
